# Combination Surgical Excision and Fractional Carbon Dioxide Laser for Treatment of Rhinophyma

**Published:** 2012-01

**Authors:** Ramtin Kassir, Johonna Gilbreath, Ali Sajjadian

**Affiliations:** Department of Plastic Surgery, Hoag Presbytarian University Hospital, Newport Beach, CA, USA.

**Keywords:** Surgical Excision, Fractional CO2 Laser, Treatment, Rhinophyma

## Abstract

Rhinophyma is a severe late complication of rosacea, which is characterized by progressive hyperplasia of sebaceous glands and connective tissue involving the lower two-thirds of the nose. It can be an emotionally devastating disorder, and serve as a medium for occult cancers and other health problems. Many surgical treatments have been advocated, as well as dermabrasion and laser therapy. In light of the problems faced with these individual therapy modalities, we advocate a combination therapy of surgical debulking and fractionated Carbon dioxide laser therapy. By excising the tissue first, we not only decreased the amount of time needed for the procedure, we were also able to preserve a histopathologic specimen that can be examined for occult cancers. After debulking, the fractionated carbon dioxide laser is then used to blend. By using fractionated carbon dioxide, we avoided the complications associated with non-fractionated carbon dioxide lasers, such as delayed healing times, hypopigmentation, hyperpigmentation, scarring, and persistent redness. We report our experience in two patients with rhinophyma who underwent a combination of surgical excision and fractional Co_2_ laser therapy for treatment. Pre- and postoperative protocols and treatment parameters are discussed. Both patients had excellent cosmetic and functional results and were followed for at least one year.

## INTRODUCTION

**Fig. 1:**
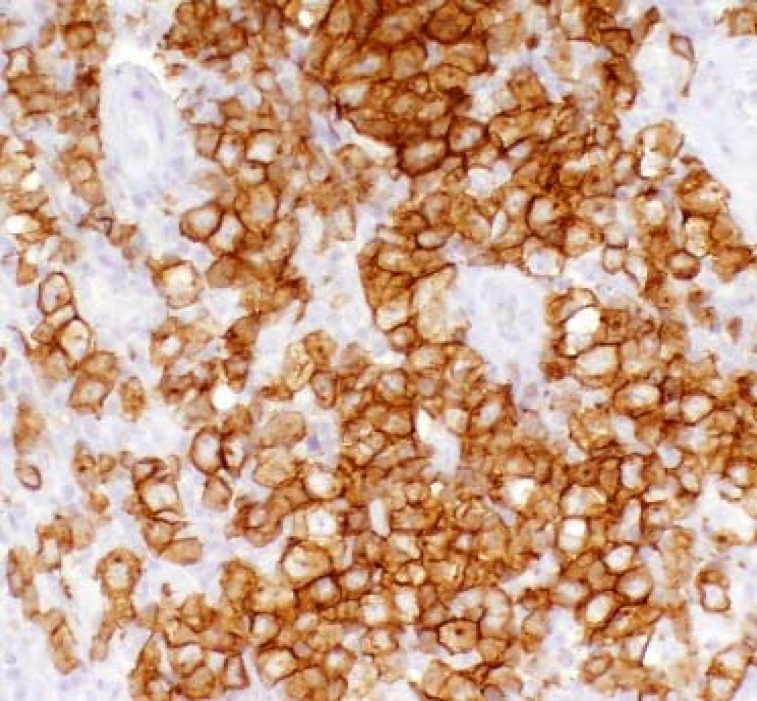
Domenico Ghirlandaio depicts a patrician with a large rhinophyma

Rhinophyma is a severe late complication of rosacea, which is characterized by progressive hyperplasia of sebaceous glands and connective tissue involving the lower two-thirds of the nose.^1^ Rosacea is typically more common in fair-skinned women aged thirty to fifty with women being affected two to three times more often than men.^2^ However, rhinophymas are almost exclusively seen in middle-aged to elderly men.^1^ This 15^th^ century painting, *An Old Man and His Grandson*, by Domenico Ghirlandaio depicts a patrician with a large rhinophyma (Figure 1).

Although rhinophyma is a benign disorder, the physiological, cosmetic and psychological aspects of the disease are great in number. Large rhinophymas can cause distortion and obstruction of the nasal cavity making breathing difficult. Possibly resulting in obstructive sleep apnea, which can lead to many other health problems.^3^ There have also been accounts of rhinophymas containing occult cancers, such as squamous cell and basal cell carcinomas.^4^ The disorder causes progressive disfiguration which can be anxiety-provoking and disturbing to the patient. Patients may become embarrassed or depressed about their appearance, sometimes resorting to reclusive behavior.^2^ Historically, rhinophymas have been a source of negative stigmatism. Having a rhinophyma was considered a sign of alcoholism, as implied by the term “whiskey nose” or “rum nose.”^3^ While it is true that alcohol can cause the skin to have a flushed appearance, rhinophymas are not found solely in alcoholics.^8^

**Fig. 2:**
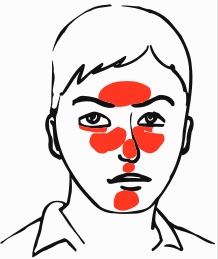
The central facial distribution of rosacea

The diagnosis of rhinophyma is based on clinical presentation and a history of rosacea. While there is not a specific diagnostic test for rosacea, classification systems exist. In 1994, Wilkin outlined four stages of rosacea (prerosacea, vascular rosacea, inflammatory rosacea and late rosacea). Recurrent flushing and blushing of the face is considered prerosacea.^5^ Blushing may occur as a result of sun exposure, emotional stress, alcoholic beverages, hot beverages, spicy food, exercise, or extreme hot or cold weather.^2^ Vascular rosacea is the second stage and is considered the first definitive stage of rosacea, which consists of persistent erythema, edema, telangiectasia and ocular rosacea. The third stage is inflammatory rosacea and consists of papules and pustules. The final stage is rhinophyma.^5^ It is important to note that rosacea can present at any stage and may not progress to later stages (Figure 2).^6^

In 2002, the National Rosacea Society (NRS) took Wilkin’s criteria a step further in an attempt to standardize the diagnosis of rosacea. The NRS described primary and secondary diagnostic features of rosacea and defined four subtypes and one variant. The diagnosis of rosacea requires the presence of one or more primary features in a central facial distribution and may include one or more secondary features. Primary features include transient erythema (blushing), non-transient erythema (persistent redness), papules and pustules, and telangiectasia. Secondary features include facial skin hypersensitivity (burning or stinging sensations), plaques, dry appearance, edema, ocular manifestations, peripheral (non-facial) locations and phymatous changes. The subtypes included erythematotelangiectatic, papulopustular, phymatous, and ocular. Rhinophyma is the most common presentation of phymatous rosacea and may be observed following or in combination with the erythematotelangiectatic and papulopustular subtypes.^6^

The NRS also established a standard grading system for rosacea and phymatous changes. Severity of rhinophyma is rated from 0 to 3, with 0 being absent, 1 being patulous follicles but no contour changes, 2 being a change in contour without nodular component, and 3 being a change in contour with a nodular component.^7^

Treatment for rosacea is generally nonsurgical and aimed at controlling the disorder rather than curing it. Topical and oral antibiotics and retinoids have been shown effective for this purpose. However, there is no conclusive evidence showing that medication alone can cause regression of rhinophyma.^1^ Invasive techniques remain the best treatment modality. 

## CASE REPORT

We report our experiences with two patients. Our first patient was a 52 years old male with a history of rosacea, rhinophyma, facial telangiectasia and obesity. The patient sought treatment for a rhinophyma that was causing nasal obstruction and nasal deformity. The patient had excellent cosmetic results post-surgery and was further treated in our office for telangiectasias. The patient began IPL treatment for rosacea one month after the initial surgery and received six total treatments.

Our second patient was a 73 years old male that was seen in our office for a rhinophyma with significant nasal deformity that was causing him emotional distress. He had a history of hypertension and diabetes mellitus. He was also recently diagnosed with a malignant melanoma one month prior to his initial consultation. We excised the melanoma and rhinophyma during the same visit. The pathology report from the nasal lesion was consistent with rhinophyma and showed sebaceous hyperplasia with multiple small epidermal inclusion cysts. Photos were taken before surgery and two months after surgery. The patient was followed for five months total and maintained excellent cosmetic and functional results.

Both patients underwent combination therapy with surgical excision of their rhinophyma followed by fractionated carbon dioxide laser resurfacing. The entire procedure was done in the office for Patient 1. Patient 2 had his procedure done in the operating room, since he required sedation for the melanoma excision. For patient 2, carbon dioxide laser resurfacing took place in the office. 

The patients were prepped and draped in a sterile fashion, 1% lidocaine with 1:100,000 dilution of epinephrine was used for anesthesia of the nose. After adequate time for vasoconstriction, a 10 blade was used to plane the nose down of overgrown sebaceous glands and achieve proper nasal contour. Then, the carbon dioxide laser was set at 30 watts, 4 msec pulse duration, 90% coverage with an 18 mm spot was used to resurface the remaining skin. Bacitracin ointment was placed on the nose. Total estimated blood loss for the procedure was less than 30 ml for patient 1 and less than 100 ml for patient 2. Both patients tolerated the procedure well.

**Fig. 3:**
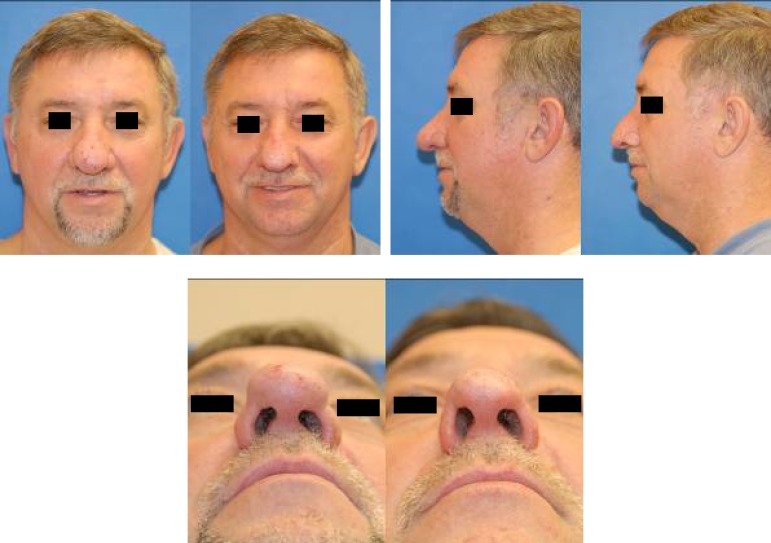
Patient 1 before and 24 weeks after treatment

**Fig. 4:**
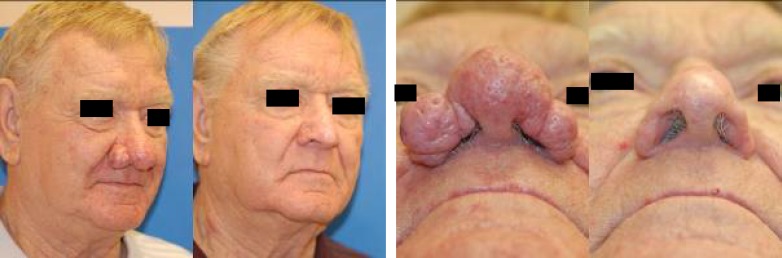
Patient 2 before and three months after treatment

Both patients were followed up at one day, one week, 3 weeks, 6 weeks, 24 weeks, 3 months and 6 months postoperatively. The nasal dorsum was re-epithelialized by 7 days in both cases. Erythema persisted for 2-3 weeks and had completely faded by one month. Both patients reported improved breathing and satisfaction with cosmesis (Figure 3 and 4). 

## DISCUSSION

Rhinophyma cause marked cosmetic, psychologic and physiologic problems for the patients. They wish to solve it in an effective way. Many surgical treatments have been advocated, as well as dermabrasion and laser therapy. However, all treatments have their advantages and disadvantages. Scalpel excision, dermabrasion and cryosurgery are all fast procedures that offer ease in handling, however, they provide little to no hemostasis.^1^ The Argon laser allows for selective coagulation of blood capillaries, but has many disadvantages, most importantly is the uncontrolled depth of tissue destruction.^1^ The carbon dioxide laser offers cosmetic precision, but when used alone can become a very time consuming method.^1^ Carbon dioxide lasers have also been shown to cause hypo or hyper-pigmentation, scarring and persistent redness.^1^^,^^9^ Electrocautery is a fast procedure with good hemostasis, but is less precise and has a high probability of scarring.^1^

We advocate a combination therapy of surgical debulking and fractionated carbon dioxide that minimizes the risks and complications of the treatments and affords the best short and long term results. The procedure can typically be done on an outpatient basis using local anesthesia, rather than general anesthesia. In the first step, we recommend surgically debulking the rhinophyma using a number 10 blade scalpel to ensure proper contour of the nose. By excising the tissue, we were able to preserve a histopathologic specimen that can be examined for occult cancers. After debulking, the fractionated carbon dioxide laser was used to blend. By using fractionated carbon dioxide, we avoided the complications associated with non-fractionated carbon dioxide lasers, such as hypopigmentation, hyperpigmentation, scarring, and persistent redness.

The carbon dioxide laser alone provides excellent cosmetic results, but is a time consuming technique, which is why we recommend debulking the lesion first to reduce operating time. Also, the carbon dioxide laser vaporizes the tissue, leaving no available tissue for histopathologic examination. Overall, the use of combination excision and fractional carbon dioxide laser allows for a much faster treatment and reduced healing time for the patient.

## CONFLICT OF INTEREST

The authors declare no conflict of interest.
